# Investigating the causal relationship between immune cell and Alzheimer’s disease: a mendelian randomization analysis

**DOI:** 10.1186/s12883-024-03599-y

**Published:** 2024-03-18

**Authors:** Min Shen, Linlin Zhang, Chen Chen, Xiaocen Wei, Yuning Ma, Yuxia Ma

**Affiliations:** Shandong University of Traditional Chinese Medicine, Jinan, Shandong Province China

**Keywords:** Alzheimer’s disease, Immune cell, Causal inference, Brain, MR analysis, Sensitivity

## Abstract

**Background:**

Complex interactions between the immune system and the brain may affect neural development, survival, and function, with etiological and therapeutic implications for neurodegenerative diseases. However, previous studies investigating the association between immune inflammation and Alzheimer’s disease (AD) have yielded inconsistent results.

**Methods:**

We applied Mendelian randomization (MR) to examine the causal relationship between immune cell traits and AD risk using genetic variants as instrumental variables. MR is an epidemiological study design based on genetic information that reduces the effects of confounding and reverse causation. We analyzed the causal associations between 731 immune cell traits and AD risk based on publicly available genetic data.

**Results:**

We observed that 5 immune cell traits conferred protection against AD, while 7 immune cell traits increased the risk of AD. These immune cell traits mainly involved T cell regulation, monocyte activation and B cell differentiation. Our findings suggest that immune regulation may influence the development of AD and provide new insights into potential targets for AD prevention and treatment. We also conducted various sensitivity analyses to test the validity and robustness of our results, which revealed no evidence of pleiotropy or heterogeneity.

**Conclusion:**

Our research shows that immune regulation is important for AD and provides new information on potential targets for AD prevention and treatment. However, this study has limitations, including the possibility of reverse causality, lack of validation in independent cohorts, and potential confounding by population stratification. Further research is needed to validate and amplify these results and to elucidate the potential mechanisms of the immune cell-AD association.

**Supplementary Information:**

The online version contains supplementary material available at 10.1186/s12883-024-03599-y.

## Introduction

Alzheimer’s disease (AD) is a prevalent neurodegenerative disorder characterized by progressive impairment of memory, cognition, and behavior, resulting in severe deterioration of patients’ quality of life and social function [1, 2]. Globally, approximately 50 million people are affected by AD or other forms of dementia [3]. The current pharmacological interventions employed for AD, namely cholinesterase inhibitors and NMDA receptor antagonists, primarily offer symptomatic relief without exerting any substantial influence on the underlying pathological processes of neuronal degeneration and demise. These treatments are incapable of modifying or reversing the progressive neurodegenerative cascade associated with the disease [4, 5]. Hence, it is crucial to identify modifiable risk factors and preventive strategies for AD.

Early-life exposure to various stimuli, such as infections, trauma, stress, and others, can trigger peripheral immune responses that are associated with neurodegeneration in later life, as evidenced by several epidemiological studies [6–8]. The intricate interplay between the immune system and the brain, which affects neural development, survival, and function, may have etiological and therapeutic implications for neurological disorders such as AD. Cytokines, which are essential mediators of infection and inflammation, participate in the bidirectional communication between the brain and the immune system [9]. Patients with AD exhibit altered levels of various cytokines in their blood and cerebrospinal fluid, indicating a chronic low-grade systemic inflammation. Moreover, different immune cells, such as macrophages, microglia, T lymphocytes, and B lymphocytes, play a role in the pathogenesis of AD [10–12]. These cells modulate amyloid-β (Aβ) clearance, neuronal viability, synaptic plasticity, and other mechanisms [13].

Recent studies have revealed that immune regulation influences the development of AD through multiple pathways, such as epigenetic modifications, checkpoint molecules, neurotrophic factors, and adrenergic signaling [14–18]. Epigenetic mechanisms, such as DNA methylation, histone modifications, and noncoding RNAs, regulate the expression of genes involved in immune responses and neurodegeneration [19, 20]. Checkpoint molecules, such as programmed cell death protein 1 (PD-1) and cytotoxic T-lymphocyte-associated protein 4 (CTLA-4), are involved in regulating immune responses and preventing excessive immune activation [21]. Neurotrophic factors, such as nerve growth factor (NGF) and brain-derived neurotrophic factor (BDNF), are secreted by immune cells and affect neuronal survival, differentiation, and function [22]. Adrenergic signaling, mediated by the sympathetic nervous system and catecholamines, modulates the activity and phenotype of immune cells and influences the inflammatory milieu in the brain [23]. These studies suggest that immune regulation is a key factor in the pathophysiology of AD and a potential target for therapeutic intervention.

However, the relationship between immune cells and AD has been inconclusive across previous studies, potentially due to methodological challenges such as small sample sizes, inadequate experimental design, and confounding variables [24, 25].

Mendelian randomization (MR) is an analytical technique based on the principles of Mendelian genetics, mainly used for causal inference in epidemiology. It utilizes genetic variation as an instrumental variable (IV) for risk factors. To ensure valid IVs for causal inference, three core assumptions must be satisfied: (1) a direct association between genetic variation and exposure, (2) no association between genetic variation and potential confounders between exposure and outcome, and (3) no influence of genetic variation on outcomes through pathways other than exposure [26–29]. Previous observational studies have identified numerous immune cell characteristics associated with AD [30–33]. In this study, we performed a comprehensive two-sample MR analysis to examine the causal relationship between 731 immune cell characteristics and AD.

The primary objective of this study is to investigate the causal relationship between immune cell variations and the risk of Alzheimer’s disease using Mendelian randomization analysis. We aim to overcome the limitations of previous observational studies by leveraging genetic variation as an instrumental variable, thereby providing more robust evidence for the role of immune cells in the pathogenesis of AD.

## Materials and methods

### Study design

Mendelian randomization (MR) is an analytical method that uses genetic variants, known as instrumental variables (IVs), to establish causal relationships between an exposure (risk factor) and an outcome. This method relies on three key assumptions: (a) the chosen genetic variants (or single nucleotide polymorphisms, SNPs) are associated with the exposure, (b) they are not associated with confounders, and (c) they influence the outcome only through the exposure, not through other pathways [34].

### Data sources and selection of instrumental variables

We used summary statistics from a genome-wide association study (GWAS) for AD from the FinnGen project (R9), which involved 9301 cases and 367,976 controls of European ancestry. We also sourced summary statistics of immune-related GWAS from the GWAS Catalog (accession numbers GCST90001391 to GCST90002121) [35]. Both datasets were of European populations, ensuring consistency in our analysis.

For the selection of instrumental variables, we first chose SNPs at a genome-wide significance threshold of *P* < 1 × 10^− 5^. Next, we ensured independence of the instruments by applying a linkage disequilibrium clumping procedure with a stringent cut-off r^2^ = 0.001 and distance = 10,000 kb. Lastly, we only retained SNPs that were more strongly associated with the exposure than the outcome (P_exposure_ < P_outcome_), to avoid direct associations with the outcome.

### Statistical analyses

We applied multiple MR methods, including inverse-variance weighted (IVW), weighted mode, sample mode, weighted median, and MR Egger. Each of these methods has specific strengths, and together they provide a comprehensive analysis:(1). IVW: This is the primary method, which uses all the genetic variants to estimate the causal effect. (2). Weighted mode: Gives more weight to the most frequently observed causal estimate, useful when a subset of instruments is valid. (3). Sample mode: Uses a simple count rather than weighting, providing a robust estimate when a mode exists. (4). Weighted median: Balances precision with robustness to invalid IVs, providing a valid causal estimate even if up to 50% of the information comes from invalid instruments. (5). MR Egger: Tests for and adjusts for pleiotropy, or the influence of genetic variants on multiple traits [36–40].

We used MR-PRESSO to detect and correct outliers in IVW linear regression and assessed heterogeneity using the Cochran Q test. If the Cochran Q test *p*-value was greater than 0.05, indicating no significant heterogeneity, we used the fixed-effects IVW method; otherwise, we used the random-effects IVW method. The analyses were performed using the Two Sample MR and MR-PRESSO packages in R [38].

## Results

### Immune cell types that confer protection against AD

**Fig. 1:**
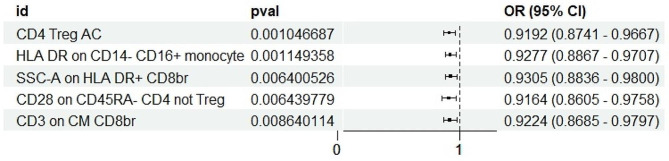
Immune cell types that confer protection against AD

We performed IVW (*P* < 0.01) as the main analysis, and the results indicated that 5 immune cells had a protective effect on AD (Fig. 1). We observed that the following immune cells were inversely associated with AD risk: CD28 on CD45RA- CD4 not Treg (0.9164, [0.8605–0.9758], 0.0064), CD3 on CM CD8br (0.9224, [0.8685–0.9797], 0.0086), CD4 Treg AC (0.9192, [0.8741–0.9667], 0.0010), HLA DR on CD14- CD16 + monocyte (0.9277, [0.8867–0.9707], 0.0011), SSC-A on HLA DR + CD8br (0.9305, [0.8836-0.9800], 0.0064).

### Immune cell types that increase the risk of AD

**Fig. 2:**
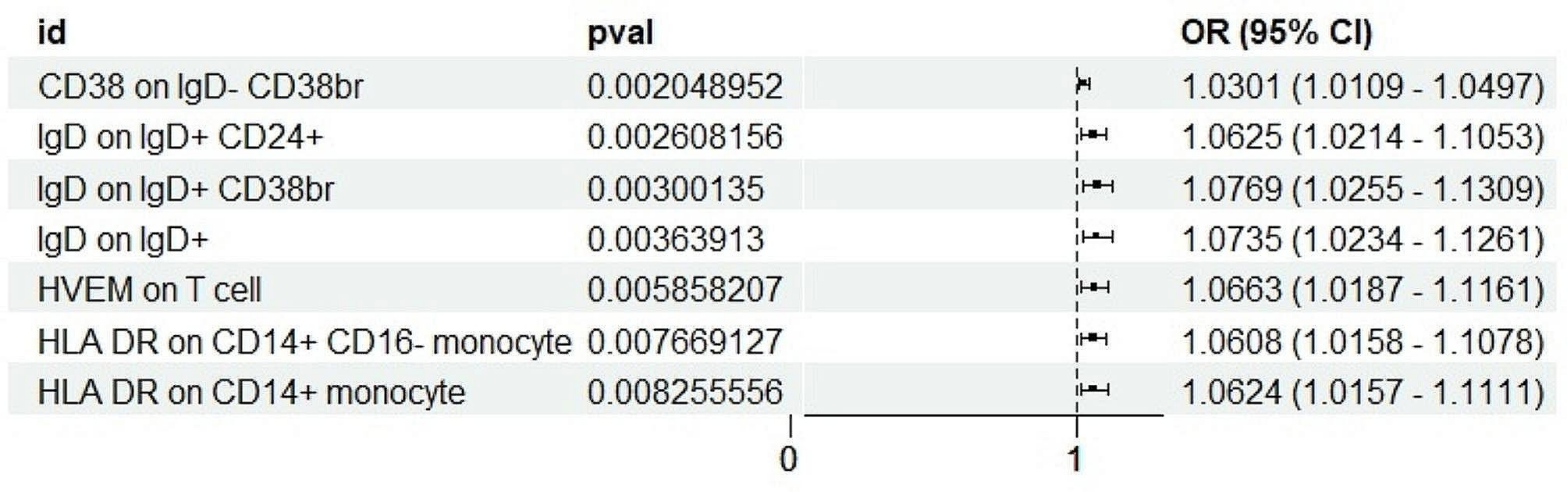
Immune cell types that increase the risk of AD

We performed the main analysis using the IVW method(*P* < 0.01), which revealed that 7 immune cell markers were genetically predicted to be positively associated with AD: CD38 on IgD- CD38br (1.0301, [1.0109–1.0497], 0.0020),HLA DR on CD14 + CD16- monocyte (1.0608, [1.0158–1.1078], 0.0077),HLA DR on CD14 + monocyte (1.0624, [1.0157–1.1111], 0.0083), HVEM on T cell (1.0663, [1.0187–1.1161], 0.0059), IgD on IgD + CD24+ (1.0625, [1.0214–1.1053], 0.0026),IgD on IgD + CD38- unsw mem (1.0651, [1.0138–1.1190], 0.0123),IgD on IgD + CD38br (1.0769, [1.0255–1.1309], 0.0030) (Fig. 2).

### Sensitivity analysis

We assessed the presence of horizontal pleiotropy, which occurs when some genetic variants used as IVs have direct effects on the outcome that are not mediated by the exposure, using the MR-Egger intercept and MR-PRESSO methods. We also evaluated the heterogeneity of causal estimates across IVs using the Cochran Q statistic in the MR-Egger method. We considered evidence of pleiotropy or heterogeneity if the *P* value was lower than 0.05 (Table 1). In addition, we conducted a leave-one-out sensitivity analysis to detect any influential IVs on the main results and ensure the robustness of the MR results. The results of the pleiotropy and sensitivity analysis are presented in Table 1. The overall direction of the results of several other sensitivity analyses agreed with the IVW point estimates. We did not detect heterogeneity or horizontal pleiotropy using Cochrane Q-analysis and MR-PRESSO global tests, respectively, indicating that the IVW results were reliable (Table 1).

**Table 1:** Heterogeneity and pleiotropy analysis of immune cell with AD using different analytical methods

Exposure	Cochran Q statistic (MR Egger)	Heterogeneity *p*-value (MR Egger)	Pleiotropy *p*-value (MR Egger)	PRESSO global outlier test	
				RSSOBs	*p*-value (Robust adjusted Profile Score)
CD4 Treg AC	11.090	0.921	0.936	12.467	0.948
CD38 on IgD- CD38br	8.512	0.744	0.242	10.431	0.804
IgD on IgD + CD24+	28.109	0.255	0.940	32.406	0.303
IgD on IgD + CD38br	26.647	0.225	0.078	34.551	0.162
IgD on IgD+	16.074	0.652	0.104	24.573	0.446
CD3 on CM CD8br	10.055	0.758	0.328	12.235	0.8
HVEM on T cell	23.258	0.330	0.649	26.114	0.397
CD28 on CD45RA- CD4 not Treg	7.822	0.729	0.788	8.897	0.847
HLA DR on CD14- CD16 + monocyte	13.644	0.692	0.584	14.712	0.803
HLA DR on CD14 + CD16- monocyte	22.086	0.336	0.803	24.543	0.431
HLA DR on CD14 + monocyte	21.405	0.315	0.810	23.895	0.383
SSC-A on HLA DR + CD8br	24.474	0.323	0.309	28.438	0.343

## Discussion

Alzheimer’s disease (AD) is a neurodegenerative disorder characterized by progressive cognitive impairment, memory loss, and behavioral changes [1, 2]. The pathogenesis of AD is complex and multifactorial, involving genetic, environmental, and immunological factors [41]. Previous studies have indicated that immune cells are involved in modulating the inflammatory and immune responses in the brain, which may influence the amyloid-beta (Aβ) deposition, tau phosphorylation, neuronal damage, and synaptic dysfunction in AD [42, 43].

Our study employed an exhaustive Mendelian randomization analysis to explore potential causal associations between 731 immune cell phenotypes and the risk of Alzheimer’s Disease (AD). Our analysis revealed that out of these, five immune cell traits appear to confer protection against AD, while seven immune cell traits were associated with an increased risk. The implicated immune cell traits spanned a broad range of T cells, B cells, monocytes, myeloid cells, and dendritic cells, underscoring the multifaceted and complex role of the immune system in AD pathogenesis. Our data suggest that both components of the immune response, namely innate and adaptive immunity, play significant roles in the etiopathogenesis of AD. Furthermore, different immune cell subsets may have unique or even opposing roles in modulating inflammation and immunity within the brain.

The protective immune cell traits against AD encompassed CD4 Treg AC, HLA DR on CD14- CD16 + monocyte, SSC-A on HLA DR+, CD8br CD28 on CD45RA- CD4 not Treg, CD3 on CM CD8br. These traits may mitigate the risk of AD through various mechanisms, potentially including inflammation suppression, augmentation of Aβ clearance, stimulation of neurogenesis, or maintenance of synaptic plasticity. For example, Tregs are recognized for their anti-inflammatory and immunoregulatory functions, which they exert by secreting cytokines like IL-10 and TGF-β, expressing molecules such as CTLA-4 and PD-1, or directly killing effector T cells [44]. Prior research has shown that enhancing the quantity or function of CD4 Tregs can ameliorate AD pathology and cognitive impairment [45–47].

In addition, classical monocytes, alternatively labeled as HLA DR + CD14- CD16 + monocytes, differentiate into macrophages or dendritic cells, and execute phagocytosis. They also participate in antigen presentation and immune regulation [48]. Earlier studies have indicated that these monocytes can protect against neurodegeneration by enhancing Aβ clearance and promoting neurogenesis [49, 50].

Our analysis identifies specific subgroups of immune cells including CD38 on IgD- CD38br, IgD on IgD + CD24+, IgD on IgD + CD38br,IgD on IgD+, as potential risk factors for Alzheimer’s disease through Mendelian randomization analysis. These immune cell subsets may play crucial roles in neuroinflammation and neuronal damage, both of which are known to be intimately linked with the pathogenesis of Alzheimer’s disease [51–53].

Our study has some limitations that should be considered. First, we applied a two-sample MR approach based on publicly available summary statistics from large GWAS cohorts. Therefore, we were unable to perform stratified analyses by sex, age, or other factors that may modify the associations between immune cell traits and AD risk. Second, we used a European ancestry population as the reference panel for both exposure and outcome GWAS. Therefore, our results may not be applicable to other ethnic groups or populations with different genetic backgrounds. Third, we used a relatively lenient threshold for selecting instrumental variables for each immune cell trait to increase the statistical power and avoid weak instrument bias. However, this may also increase the likelihood of false positives or horizontal pleiotropy. Fourth, we could not account for the potential interactions or synergies among different immune cell traits or other factors that may influence the immune system in AD. Therefore, our results should be interpreted with caution and validated by further studies.

We proposed some future directions and implications for further research. Future studies should include more immune cell types, larger and more diverse populations, more biomarkers, and functional experiments to elucidate the causal mechanisms of immune system and AD. Our results offer valuable insights for the prevention and treatment of AD by modulating the immune system.

## Future aspect

The findings of this study open up new avenues for future research in Alzheimer’s disease. Understanding the distinct roles of different immune cell subsets in AD can pave the way for the development of novel therapeutic strategies that modulate the immune response to combat neurodegeneration. Future studies could focus on exploring the mechanisms through which these immune cell traits influence AD pathogenesis and progression. In addition, the potential therapeutic effects of enhancing the protective immune cell traits or inhibiting the harmful ones could be investigated in preclinical and clinical trials. Furthermore, the use of high-throughput technologies and multi-omics approaches can provide a more comprehensive understanding of the immune landscape in AD. Ultimately, these efforts could contribute to the development of personalized immunotherapies for AD, improving patient outcomes and quality of life.

## Conclusion

Our study findings suggest that immune regulation is crucial for the pathogenesis of AD and provide novel insights into the potential targets for prevention and treatment of AD. The results were consistent across various sensitivity analyses that evaluated pleiotropy and heterogeneity. However, some limitations of this study should be acknowledged, such as the potential confounding by population stratification, the lack of validation in independent cohorts, and the possibility of reverse causation.

### Electronic supplementary material

Below is the link to the electronic supplementary material.

Supplementary Material 1

## Data Availability

Data is provided within the manuscript or supplementary information files.
